# Does glycaemic control or renal function provide a better indicator for the service support required in type 1 diabetes?

**DOI:** 10.1111/dme.70050

**Published:** 2025-05-01

**Authors:** Adrian H. Heald, Mike Stedman, Edward Jude, Hellena Habte‐Asres, Angus Forbes, Martin Whyte, J. Martin Gibson, William Ollier

**Affiliations:** ^1^ The School of Medicine and Manchester Academic Health Sciences Centre Manchester University Manchester UK; ^2^ Department of Endocrinology and Diabetes Salford Royal Hospital Salford UK; ^3^ Res Consortium Andover UK; ^4^ Department of Diabetes Tameside General Hospital Greater Manchester UK; ^5^ Department of Diabetes Kings College Hospital London UK; ^6^ Department of Clinical and Experimental Medicine University of Surrey Guildford UK; ^7^ Faculty of Science & Engineering Manchester Metropolitan University Manchester UK

**Keywords:** eGFR, HbA_1c_, outpatient appointments, type 1 diabetes, urine ACR

Advances in blood glucose measurement and insulin dosing used in the management of type 1 diabetes (T1D) over the last 25 years have provided a frameshift of opportunity for improving outcomes relating to how clinical monitoring data are derived, and the perceptions that patients have regarding their care strategies to achieve therapeutic goals.^1^

Chronic kidney disease (CKD) in people with T1D can be slowed or prevented through the use of renal protective therapies such as antagonists of the renin‐angiotensin system.^2^ However, CKD remains a continuous and major management challenge, as does maintaining blood glucose levels within the target range. The number of specialist appointments attended each year and required by each patient might be used as a measure of the overall health status of the individual.^3^ This study aimed to determine whether glycaemic control (HbA_1c_) or renal function [estimated glomerular filtration rate (eGFR) and albumin creatinine ratio (ACR)] is a better predictor for the need for intensive specialist care in T1D.

Only a relatively small number of long‐term cohorts of people with T1D with longitudinal data have been described.^4^ We have been able to examine the continuous health records of T1D individuals collected for over 15 years (2008–2023) using the Salford (United Kingdom) Diabetes Cohort. This encompasses all individuals diagnosed as having T1D with primary and secondary care follow‐up data for up to 25 years from 2000 onwards. Ethical permission was obtained (23/WS/0175 Favourable Opinion on 8 March 2024).

Patients with over 10 years of results within the study period were split across their middle calendar year (between 2008 and 2023). For cross‐sectional analysis, the median value in each patient for HbA_1c_, eGFR, ACR, and annual number of outpatient appointments was compared to the overall median value to classify their results as either high or low. For longitudinal analysis, the median change between the first and second halves of the period 2008–2023 was applied to calculate high and low change in eGFR versus outpatient appointments. For both cross‐sectional and longitudinal analyses, the simple odds ratio linking the numbers in high and low classes of HbA_1c_, eGFR and ACR to high and low outpatients was calculated.

Six hundred and ninety‐six (696) individuals with T1D (mean age 51.4 years, *n* = 302 females) had sufficient data to analyse. We took a threshold of >4 outpatient (OP) attended appointments per year as indicative of intense input using simple linear regression as the median number of OP appointments per day was 4.1.

There was no link between mean level of HbA_1c_ (<65 vs. ≥65 mmol/mol) and relative number of attended OP appointments each year (OR 0.84 95% confidence interval [CI] 0.64–1.04).

In any calendar year, individuals with a lower eGFR (<60 mL/min/1.73 m^2^) had double the relative probability of having >4 appointments per year than those with a higher eGFR (odds ratio [OR] 2.9 [95% CI 2.6–3.2]). For urine albumin/creatinine ratio (ACR) ≥3.0 mg/mmol versus a normal ACR, the corresponding OR was 1.4 (95% CI 1.3–1.5) for >4 OP appointments per year. T1D individuals with a larger relative decrease in eGFR (>6.4 mL/min/1.73 m^2^ over 6 years) also showed significant increases in the likelihood of >4 appointments each year with an OR of 1.4 (95% CI 1.3–1.5) vs. those people with a less rapid decline in eGFR.

Our analysis in a cohort of people with T1D included all those who attended outpatient appointments, and it suggests that chronic kidney disease and the presence of microalbuminuria in T1D link closely with increased hospital outpatient appointment frequency and are markers for overall levels of morbidity, with attendant economic costs.^5^

A limitation of the analysis is that we are unable precisely to determine the nature of the specialty consulted for all of the outpatient appointments. We were, however, able to determine that overall 76% of outpatient appointments were at diabetes, renal or cardiology clinics.

Thus, effective prevention of renal decline, using ACR/eGFR monitoring, is as important as glycaemic control in maintaining good health as measured here by the requirement for hospital attendance, and as highlighted previously in people with T2D.^6^ Use of evidence‐based therapies for renal health should remain a priority for all clinicians in T1D management.^2^

We suggest that renal function markers (eGFR and ACR) may be stronger indicators than glycaemic control of the need for intensive specialist support in people with T1D.

## AUTHOR CONTRIBUTIONS

AHH, WO and MS conceived the study. MS led on data analysis. MG, EJ and MW provided invaluable insight in relation to the context of the study with expert contributions from AF and HH‐A and editorial oversight by MG and MW. All authors reviewed and approved the final version of the manuscript.

## FUNDING INFORMATION

No external funding was used for this study.

## CONFLICT OF INTEREST STATEMENT

None of the co‐authors have any conflict of interest.

## COMPLIANCE WITH ETHICAL GUIDELINES

WS/0175 Favourable Opinion 8 March 2024.

## Data Availability

The data that support the findings of this study are available upon request from the corresponding author. The data are not publicly available due to privacy or ethical restrictions.
